# Edge Effects in the Distribution of Coleoptera in the Forests of the Center of the European Part of Russia

**DOI:** 10.3390/insects14040371

**Published:** 2023-04-10

**Authors:** Alexander B. Ruchin, Leonid V. Egorov, Anatoliy A. Khapugin

**Affiliations:** 1 Joint Directorate of the Mordovia State Nature Reserve and National Park “Smolny”, Saransk 430005, Russia; 2 Prisursky State Nature Reserve, Cheboksary 428034, Russia; 3 Institute of Environmental and Agricultural Biology (X-BIO), Tyumen State University, Tyumen 625003, Russia

**Keywords:** abundance, insects, beetles, species diversity, Republic of Mordovia

## Abstract

**Simple Summary:**

Forest edges significantly influence the distribution of many beetles. In 2020–2022, in the Republic of Mordovia (Russia), we used beer traps to collect beetles in four sites, located on the forest edges, and in forest interiors. Eight traps were on each site (edge–below, edge–above, forest interior–below, and forest interior–above) with two traps per plot. The traps were located at heights of 1.5 (below) and 7.5 m (above) above the ground. More than 13,000 specimens from 35 families were recorded. There were 13 species common to all plots, including four (*Protaetia marmorata*, *Cryptarcha strigata*, *Glischrochilus grandis*, and *Soronia grisea*) in all traps. The general pattern was the highest beetle diversity on the forest edge in the lower traps, while the total number of all species on the edges was lower. At the edges, the Shannon index was almost always higher or equal to similar values in traps in the forest interior. Based on our data, the number of saproxylic beetles prevails inside forest areas, and the highest number of them was found in the upper traps. In all plots, we found a relatively higher number of anthophilic beetles at the forest edge in the upper traps.

**Abstract:**

Forest edges, which are ecotones, have a significant impact on the spatial distribution of many Coleoptera species. This research was carried out in 2020–2022 on the territory of the Republic of Mordovia (the center of the European part of Russia). Beer traps (with a bait made of beer with sugar) were used to collect Coleoptera. Four plots were selected for the research, which differed in the composition of plants on the edges, adjacent open ecosystems, and types of forest ecosystems. The forest adjoined closely to this open ecosystem. Inside the forest interior, at 300–350 m, a control inner section of the forest with a closed canopy was selected. There were eight traps on each site: edge–below, edge–above, forest interior–below, and forest interior–above, with two traps in each plot. These traps were located at a height of 1.5 (below) and 7.5 m (above) above the ground on tree branches. In total, more than 13,000 specimens from 35 families were recorded. Cerambycidae, Nitidulidae, Curculionidae, and Elateridae had the greatest species diversity. Nitidulidae (71.6% of all individuals), Curculionidae (8.3%), Scarabaeidae (7.7%), and Cerambycidae (2.4%) predominated in total number. There were 13 species common to all plots. At the same time, only four species were found in all traps (*Protaetia marmorata*, *Cryptarcha strigata*, *Glischrochilus grandis*, and *Soronia grisea*). The abundance of *P. marmorata* on all plots at an altitude of 7.5 m on the edges was greater. *G. grandis* prevailed in the lower traps. The abundance of *C. strigata* and *S. grisea* varied depending on the location of the trap on different plots. The general pattern was the greatest species diversity of Coleoptera on the edges in the lower traps. At the same time, the total number of all species on the edges was lower. At the edges, the Shannon index was practically always higher or equal to similar indicators in traps located in the forest interior. According to the average values of all plots, it turned out that the number of species of saproxylic Coleoptera prevails inside forest areas, and the largest number of saproxiles was found in the upper traps. An interesting feature for all plots was a more significant relative number of anthophilic species at the edge in the upper traps.

## 1. Introduction

Edge effects are associated with changes in abiotic conditions, such as temperature, wind, solar insolation (location of edges in relation to the Sun), and humidity, as well as with some biological reactions, such as abundance, migration, food resources, and distribution of species [1,2,3,4]. Edges in most forests represent a sharp transition between two relatively homogeneous habitats (on the horizon—a meadow, an agroecosystem, a clearing, and a forest; vertically—undergrowth and canopy). At the same time, the horizontal transition at the edges is usually sharper. The part of the habitat with the observed marginal effect is the marginal zone. The size of the edges deep into the forest may vary depending on the size of the adjacent non-forest habitat, the density of the stand, the presence of herbaceous vegetation in the forest, and other circumstances [5,6]. The strength of the edge effects decreases with walking deeper into the forest. The factors that may contribute to the variability of the edge effect include the age of the edges, the type of edge, the type of stand, the combined impact of several neighboring edges, the structure of adjacent vegetation, seasonality, the influx of animals or plants from surrounding areas, fires, and other weather phenomena [7,8,9,10].

The study of the biological diversity of different types of edges provides a clear picture of the edge effects. Four fundamental phenomena were identified that explain changes in the species abundance at the edges of habitats: ecological flows, access to spatially separated resources, resource tracking, and species interaction [9]. Coleoptera, as one of the numerous orders of insects, has long been studied in fringe biotopes. For example, Allison et al. [11] identified Cerambycidae species as the most numerous at the edges, other species are more numerous in clearings, and a third species are more often caught in the depths of the forest. At the community level, most dung beetle species demonstrated either avoidance of borders or preference. However, the response depended on the differences in the environment between the places of use (forest plantations and local forests) and ranged from a neutral reaction on mature plantations to avoiding edges on newly created forest plantations [12]. Two species of bark beetles (*Hylurgops palliatus* (Gyllenhal, 1813) and *H. glabratus* (Zetterstedt, 1828)) infected trees in the greatest number in the depths of the forest than at the edge [13]. Forest species of Carabidae are able to penetrate deep into pastures at a distance of up to 30 m from the edge of the forest. Beyond this distance, the pasture becomes a barrier for them [14]. On the other hand, the natural edges of the forest were impervious to small species of Carabidae, preventing their settlement in the depths of the forest, while both medium and large species penetrated through these edges and dispersed in the depths of the forest. Anthropogenic boundaries, supported by ongoing anthropogenic interference, were permeable to ground beetles of all sizes, which allowed them to penetrate into the forest interior [15].

In most studies that consider the edge effects on the number of Coleoptera, only one gradient is considered, for example, the inhabitants of the ground level or grassy. At the same time, such studies are carried out using standard methods such as pitfall traps, window traps, or Lindgren traps [3,11,16,17,18]. On the other hand, using different types of traps at the same time, as well as using nonconventional methods of catching with baits, provides good and unique results. In addition, the location of traps not only in a horizontal gradient (in soil, grass, or undergrowth) but also the use of the vertical installation of traps (at the level of undergrowth and crown) influence the results of research [19,20,21,22,23].

The purpose of this study was to determine the edge effects on the abundance and species diversity of Coleoptera. The objectives of the study were to (1) determine biodiversity and abundance on forest edges and inside the forest; (2) determine biodiversity and abundance at two heights relative to the Earth’s surface; (3) determine whether differences in open biotopes affect biodiversity and the number of beetles on the edges.

## 2. Materials and Methods

### 2.1. Study Area

**Figure 1:**
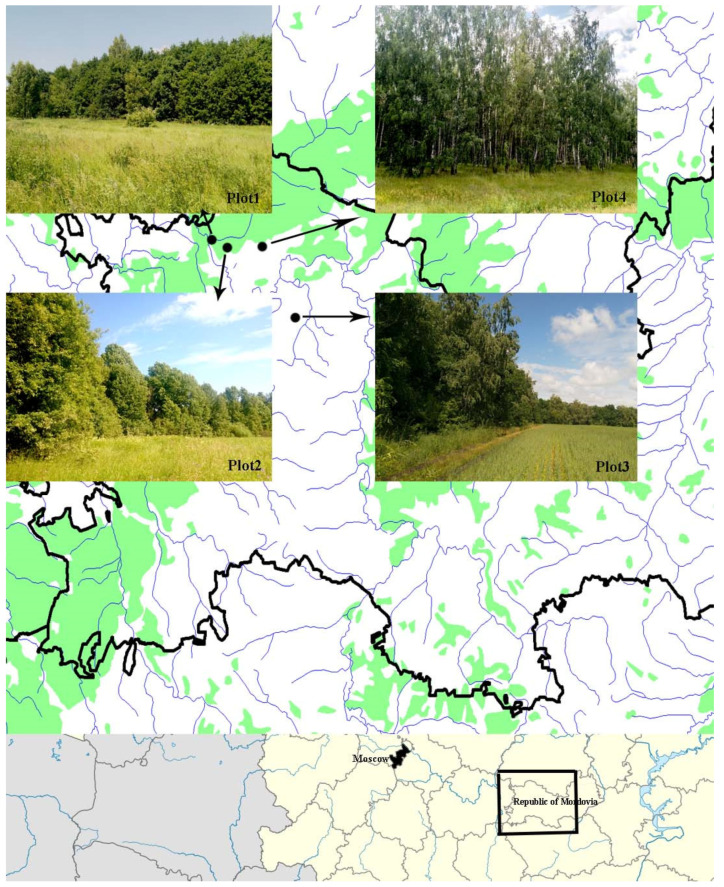
Research plots and photos of biotopes. The black box represents the study area.

All studies were conducted in the Republic of Mordovia (the center of European Russia) (Figure 1) in 2020–2022. The region is located within the Volga Upland and the Oka-Don lowland. The studies were carried out in forest ecosystems located within the Oka-Don Plain—Lowland. The vegetation cover of the plain is mainly represented by forest communities and open spaces (meadows, agroecosystems, etc.).

Four plots were studied. Each plot represented an open ecosystem (floodplain meadow, large meadow, meadow, and agroecosystem) and a deciduous forest located nearby. The forest adjoined closely to this open ecosystem. On the border (i.e., ecotone) of the open space and the forest area, a forest edge was formed. In addition, in the forest interior, at 300–350 m, a control inner plot of the forest with a closed canopy was selected. All experiments were performed simultaneously in two repetitions. Thus, there were 8 traps on each plot: edge–below, edge–above, forest interior–below, and forest interior–above, with two traps in each plot. These traps were located at a height of 1.5 (below) and 7.5 m (above) above the ground on tree branches.

At the forest edge of plot1 (54.7508 N, 43.0877 E), the crown layer (percent cover: 20%) is formed by *Quercus robur*. The shrub layer is sparse, being formed by single individuals of *Viburnum opulus*, with an undergrowth of *Ulmus* sp. The ground layer (percent cover: 60–70%) is formed by *Convallaria majalis*, *Urtica dioica*, *Cirsium arvense*, *Rubus caesius*, *Filipendula ulmaria*, *Galium aparine*, *G. boreale*, *Glechoma hederacea*, *Arctium lappa*, *Coronaria flos-cuculi*, *Heracleum sibiricum*, *Bromus inermis*, *Veronica longifolia*, *Lathyrus pratensis*, *Elymus repens*, *Vicia cracca*, *Festuca pratensis*, *Sisymbrium cheiranthoides*, *Alopecurus pratensis*, *Phleum pratense*, and *Hieracium umbellatum*. At the forest interior of plot1, the percent cover of the crown layer was 60%, being represented by *Quercus robur* (70%) and *Ulmus* sp. (30%). The second crown layer is formed by *Quercus robur*, *Tilia cordata*, and *Ulmus* sp. The shrub layer is highly sparse and represented by *Rubus idaeus* and young plants of *Quercus robur*, *Tilia cordata*, and *Ulmus* sp. The ground layer (percent cover: 80%) contains *Urtica dioica* (40%), *Glechoma hederacea* (50–60%), *Festuca gigantea*, *Arctium lappa*, *Filipendula ulmaria*, *Scrophularia nodosa*, *Ranunculus repens*, *Circaea lutetiana*, *Angelica sylvestris*, *Impatiens noli-tangere*, *Polygonum dumetorum*, and *Ranunculus cassubicus*. In the surroundings of the cordon Taratinskiy, the vegetation is formed by *Alopecurus pratensis*, *Bromus inermis*, and forbs species (*Filipendula vulgaris*, *Ranunculus polyanthemos*, *Geranium pratense*, and others). Some weed species are presented, namely, *Cirsium arvense*, *Urtica dioica*, and *Equisetum arvense*.

At the forest edge of plot2 (54.7283 N, 43.1520 E), the crown layer (percent cover: 10%) is formed by *Betula pendula*. The shrub layer is sparse, and it is represented by single individuals of *Acer platanoides* and *Rubus idaeus*, with an undergrowth of *Ulmus* sp. and *Quercus robur*. The ground layer (percent cover: 80%) contains *Festuca pratensis*, *Viscaria vulgaris*, *Phleum pratense*, *Rumex acetosella*, *Veronica officinalis*, *Hypericum perforatum*, *Galium mollugo*, *Trifolium medium*, *Elytrigia repens*, *Achillea millefolium*, *Convolvulus arvense*, *Ranunculus acris*, *Glechoma hederacea*, *Geum urbanum*, *Melampyrum pratense*, *Viola hirta*, *Clinopodium vulgare*, *Leonurus quinquelobatus*, *Lathyrus vernus*, and *Dactylis glomerata*. In the forest interior of plot2, the percent cover of the crown layer is 60%; it is formed by *Betula pendula*. The shrub layer (percent cover: 40%) includes *Frangula alnus* (10%), *Lonicera xylosteum*, *Acer platanoides* (10%), *Prunus padus*, and *Rubus idaeus*, with an undergrowth of *Ulmus* sp. (30%), *Quercus robur*, *Pinus sylvestris*, and *Tilia cordata*. The ground layer (percent cover: 70%) contains *Glechoma hederacea* (30%), *Pulmonaria obscura* (10%), *Asarum europaeum*, *Galium mollugo*, *Moehringia trinervia*, *Geum urbanum*, *Urtica dioica*, *Stellaria holostea*, *Milium effusum*, *Festuca gigantea*, *Veronica officinalis*, *Scrophularia nodosa*, *Poa nemorosa*, *Melampyrum pratense*, *Leonurus quinquelobatus*, and *Lathyrus vernus*. The large glade is located near plot2. Weeds are the main part of its vegetation. The following species have been registered: *Potentilla argentea*, *Plantago major*, *P. media*, *Elymus repens*, *Geranium pussilum*, *Agrostis gigantea*, *Dactylis glomerata*, *Geranium pratense*, and others; *Urtica dioica* and *Conium maculatum* form several thicket patches here.

At the forest edge of plot3 (54.4821 N, 43.5208 E), the crown layer (percent cover: 30%) is formed by *Quercus robur*. The second crown layer is represented by *Pinus sylvestris* and *Betula pendula*. The shrub layer is relatively dense (percent cover: 30%) and includes *Sorbus aucuparia* and an undergrowth of *Quercus robur* and *Malus domestica*. The ground layer (percent cover: 70%) contains *Galium mollugo*, *Fragaria vesca*, *Pimpinella saxifraga*, *Hypericum perforatum*, *Filipendula vulgaris*, *Viola hirta*, *Campanula trachelium*, *C. rapunculoides*, *Glechoma hederacea*, *Poa nemorosa*, *Agrimonia eupatoria*, *Galium boreale*, *Aegopodium podagraria*, *Geranium sylvestre*, *Veronica chamaedrys*, *Dactylis glomerata*, *Vicia sepium*, *Geum urbanum*, *Pyrethrum corymbosum*, *Solidago virgaurea*, and *Trifolium medium*. At the forest interior of plot3, the percent cover of the crown layer is 80%; it is formed by *Quercus robur* and *Betula pendula*. The second crown layer includes *Betula pendula* and *Larix sibirica* (artificial plantations). The shrub layer (percent cover: 50%) includes *Sorbus aucuparia*, *Prunus padus*, *Amelanchier spicata*, *Frangula alnus*, *Sambucus racemosa*, and *Viburnum opulus*, with an undergrowth of *Quercus robur*. The ground layer (percent cover: 80%) is formed by *Stellaria holostea* (40%), *Aegopodia podagraria* (20%), *Glechoma hederaceae* (8–10%), *Geum urbanum*, *Lathyrus vernus*, *Campanula rapunculoides*, *Betonica officinalis*, *Galium mollugo*, *Pimpinella saxifraga*, *Viola hirta*, *Agrimonia eupatoria*, *Carex spicata*, *Calamagrostis arundinacea*, and *Scrophularia nodosa*. Near the studied oak forest, croplands are located, being represented by winter wheat crops. On this field and along the dirt road, located nearby, the following weeds have been found: *Convolvulus arvensis*, *Plantago major*, *Sinapis arvensis*, *Lactuca serriola*, *Chenopodium album*, *Delphinium consolida,* and others.

At the forest edge of plot4 (54.7284 N, 43.3244 E), the crown layer is formed by *Betula pendula* (percent cover: 20–30%). The shrub layer is sparse and includes *Salix caprea*, *S. cinerea*, *Malus domestica*, *Frangula alnus*, and *Sorbus aucuparia*. The ground layer is well developed (percent cover: 80–85%) and includes *Phleum pratense*, *Melampyrum nemorosum*, *Leucanthemum vulgare*, *Trifolium hybridum*, *Pimpinella saxifraga*, *Seseli libanotis*, *Knautia arvensis*, *Achillea millefolium*, *Hypericum perforatum*, *Linaria vulgaris*, *Cichorium intybus*, *Erigeron annuus*, *Deschampsia cespitosa*, *Vicia cracca*, *Veronica chamaedrys*, *Angelica sylvestris*, *Lysimachia vulgaris*, *Anthoxanthum odoratum*, and *Galium mollugo*. At the forest interior of plot4, the crown layer (percent cover: 50%) is formed by *Betula pendula*. The shrub layer is sparse (percent cover: 5–10%) and includes *Sorbus aucuparia* and *Frangula alnus*, with an undergrowth of *Populus tremula*. The ground layer (percent cover: 70–80%) includes *Pimpinella saxifraga*, *Rubus saxatilis*, *Melampyrum nemorosum*, *Viola canina*, *Phleum pratense*, *Fragaria vesca*, *Plantago lanceolata*, *Hypericum perforatum*, *Dryopteris carthusiana*, *D. expansa*, *Leucanthemum vulgare*, *Platanthera bifolia*, *Agrostis gigantea*, *Galium mollugo*, *Deschampsia cespitosa*, *Convallaria majalis*, *Chamaenerion angustifolium*, and *Calamagrostis epigeios*. Near plot4, old and abandoned lands are located, where weed vegetation developed after the abandonment of arable lands approximately 30–35 years ago. Its vegetation is formed by plant communities of *Cychorium intybus*, *Agrimonia eupatoria*, *Calamagrostis epigejos*, *Artemisia campestris*, *A. vulgaris*, *Ranunculus polyanthemos*, *Cirsium arvense*, *Galeopsis* spp., *Medicago falcata*, *Euphorbia virgata*, and others.

### 2.2. Sampling Procedures

The Coleoptera was collected in the spring–autumn period of 2020–2022, when the insect activity was the highest, namely, from 7 May to 3 September in 2020, from 12 May to 20 July in 2021, and from 10 May to 22 July in 2022. In each of the four study sites, the distance between the upper and bottom traps was approximately 15–20 m. The total sampling effort was 640 exposures of traps 16 times, where at each height (on the edges or inside the forest) there were 40 repetitions. The frequency of checking the functioning of the traps and emptying them was between 7 and 15 days. The collection of the specimens was performed using traps of our own design. A five-liter plastic container with a window cut out on one side at a distance of 10 cm from the bottom was used as a trap [24]. Beer was used as bait. Honey and sugar were added to it for fermentation. The collected specimens were usually placed in plastic bags containing 70% alcohol. Then, we sorted and identified the specimens in the laboratory.

### 2.3. Identification

Within a family, the species were listed according to modern data [25,26]. They were checked in accordance with the Catalogue of Palaearctic Coleoptera [27,28,29,30,31,32,33,34,35] and other publications [36,37]. The years of description of some beetle species are indicated according to Bousquet [38].

### 2.4. Data Analyses

The analysis used data on the number of all Coleoptera individuals in the traps for the exposure time. The exposure time was the period between hanging a trap and taking specimens for analysis (expressed in days). Saproxylic species were determined taking into account indications from publications [39,40,41,42,43,44]. Species were assigned to anthophilic beetles (visiting flowers) based on our own long-term observations, as well as on the basis of publications [40,41,44,45].

To compare the species diversity of Coleoptera between the studied plots and heights, we used the Jaccard index. Based on the collected data, the widely used Shannon index and Simpson index were also calculated [46,47]. At the same time, in the calculations we did not take into account insects that were not identified at the species level.

The statistical analysis was carried out using PAST 4.07 [48]. The ordination techniques, using principal component analysis (PCA), defined the major gradients in the spatial arrangement of the studied species selected for the analysis. For the ecological interpretation of the ordination axes, groups of bait trap positions (based on the height and proximity from the forest edge) were plotted onto the PCA ordination diagram as supplementary environmental data. We analyzed the species that represented at least 100 exemplars during the sampling period. In addition, we used the coefficient of determination (R2, or R-squared) according to [49]. The Jaccard similarity index was calculated for all study plots.

## 3. Results

As a result of the material processing, 13,042 species from 35 Coleoptera families were recorded. A total of 144 species were collected and identified from all plots (Appendix A). Some species from the families Nitidulidae, Staphylinidae, Scirtidae, Throscidae, and Buprestidae could not be identified to species due to the fact of the poor preservation of materials.

The greatest species diversity was noticed in Cerambycidae (18 species), Nitidulidae (15 species), Curculionidae (14 species), and Elateridae (11 species) (Appendix A). Nitidulidae (9341 species, 71.6%), Curculionidae (1084 species, 8.3%), Scarabaeidae (1004 species, 7.7%), and Cerambycidae (309 species, 2.4%) dominated by the total number during the studies. In summary, these four families accounted for 90% of the numbers of all Coleoptera.

**Figure 2:**
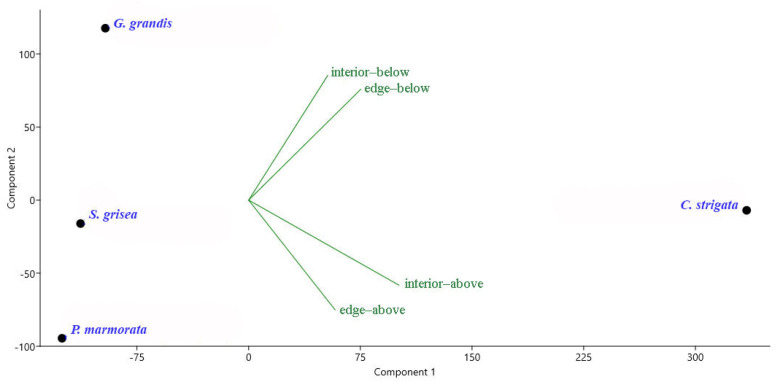
Principal component analysis (PCA) ordination diagram of some Coleoptera species based on their mean abundance at various heights of the bait trap positions (above—the height of 7.5 m, below—the height of 1.5 m) and the proximity of the forest edge (interior—inside the forest, edge—at the forest edge) in all four studied sites. Green represents bait trap positions, and purple represents four Coleoptera species. *G. grandis*—*Glischrochilus grandis*; *S. grisea*—*Soronia grisea*; *P. marmorata*—*Protaetia marmorata*; *C. strigata*—*Cryptarcha strigata*.

There were 13 species common to all plots: *Cetonia aurata* (Linnaeus, 1758), *Protaetia fieberi* (Kraatz, 1880), *Protaetia marmorata* (Fabricus, 1792), *Litargus connexus* (Geoffroy, 1785), *Cryptarcha undata* (G.-A. Olivier, 1790), *Cryptarcha strigata* (Fabricius, 1787), *Epuraea guttata* (G.-A. Olivier, 1811), *Glischrochilus grandis* (Tournier, 1872), *Glischrochilus hortensis* (Geoffroy, 1785), *Glischrochilus quadrisignatus* (Say, 1835), *Soronia grisea* (Linnaeus, 1758), *Leptura quadrifasciata* (Linnaeus, 1758), and *Anisandrus dispar* (Fabricius, 1792). However, only four species were found in all traps (*P. marmorata*, *C. strigata*, *G. grandis*, and *S. grisea*). The abundance of *P. marmorata* in all plots at an altitude of 7.5 m on the edges was greater compared to the traps located close to the soil (Figure 2). *G. grandis* prevailed in the lower traps but regardless of the edge and the interior of the forest. The abundance of *C. strigata* and *S. grisea* varied depending on the location of the traps in the different plots. Therefore, in plot1, the abundance of *C. strigata* was higher in the upper traps both at the edge and in the forest interior. However, the opposite relationship was observed in plot2. The abundance of *S. grisea* in the two plots was higher at the top on the edges, while the opposite results were obtained in the other two plots. At the same time, the average abundance of *C. strigata* and *S. grisea* for all plots did not reveal any clear dependency.

**Figure 3:**
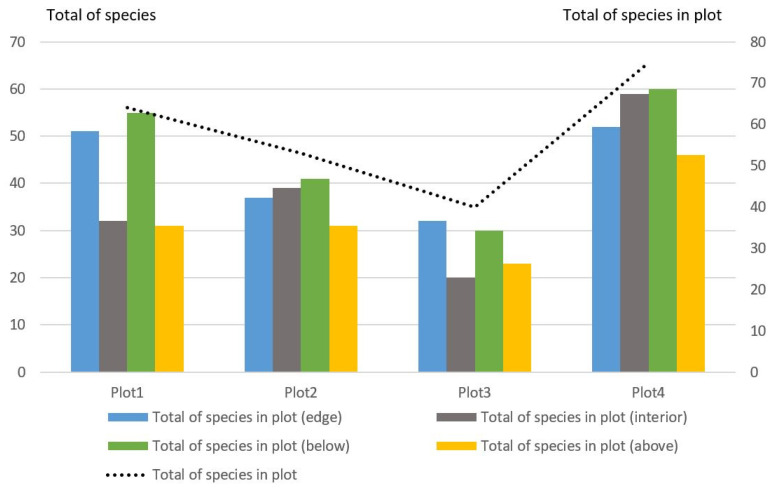
The species diversity of Coleoptera collected using beer traps on the edges and in the forest interior.

The general pattern was the greatest species diversity of Coleoptera on the edges in the lower traps. In plot1, it accounted for 64 species (Figure 3), of which 41 species (64.1%) were marked on the edge below (Table 1). In plot2, the species diversity of Coleoptera consisted of 53 species, of which 28 species (52.8%) were noted at the edge below. In plot3, the species diversity of Coleoptera included 40 species, of which 23 species (57.5%) were noted at the edge below. In plot4, the species diversity of Coleoptera numbered 75 species, of which 42 species (56.0%) were noted at the edge below.

In contrast to the species diversity, the total number of all Coleoptera species on the edges was lower. Thus, in the edge biotopes in the lower traps, the abundance was everywhere less than at a similar height in the forest interior. However, in plot4, the Coleoptera numbers were 18% lower inside the forest below (Table 1). In the upper traps on the edges, the number was also everywhere less than the inside (except plot1).

The number of Coleoptera families in the plots differed (Table 1). In addition, the number of families on the edges and inside the forest varied. This indicator, in most cases, increased in the lower traps on the edges.

At the edges, the Shannon index was almost always higher or equal to similar indicators in traps located inside the forest. The values of the Simpson index showed the opposite dependence, although not as obvious (Table 1). The calculated Shannon and Simpson indices showed the following results: the maximum values of the Shannon index were obtained in plot1 and plot4. The same plots had minimal values of the Simpson index. The lowest value of the Shannon index and the highest value of the Simpson index were obtained in plot3 (Table 1).

**Figure 4:**
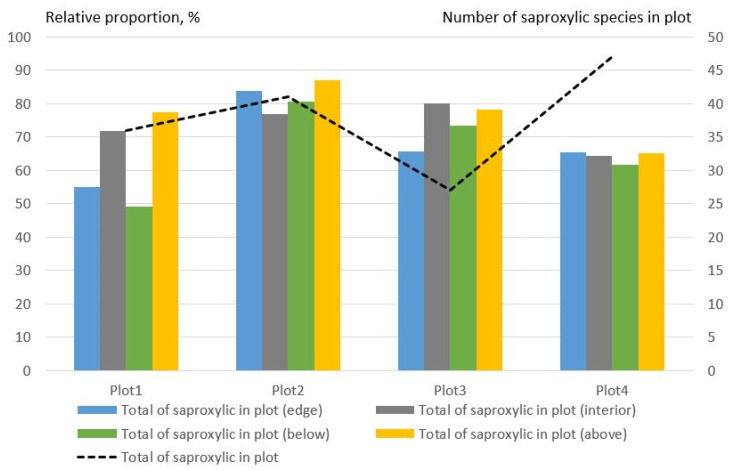
The relative proportion of saproxylic Coleoptera species collected using beer traps on the edges and in the forest interior (% of the number of species in the plot).

The proportion of saproxylic beetle species was very low in plot 1 at the edge of the lower trap. In other traps, the proportion of saproxylic species was higher than 60%. In plot4, the proportion of saproxylic species in all traps was approximately the same (Figure 4). The number of saproxylic species varied in different plots. However, according to the average data from all plots, the number of saproxylic Coleoptera species prevails in the forest interior, and the largest number of saproxylic was found in the upper traps.

**Figure 5:**
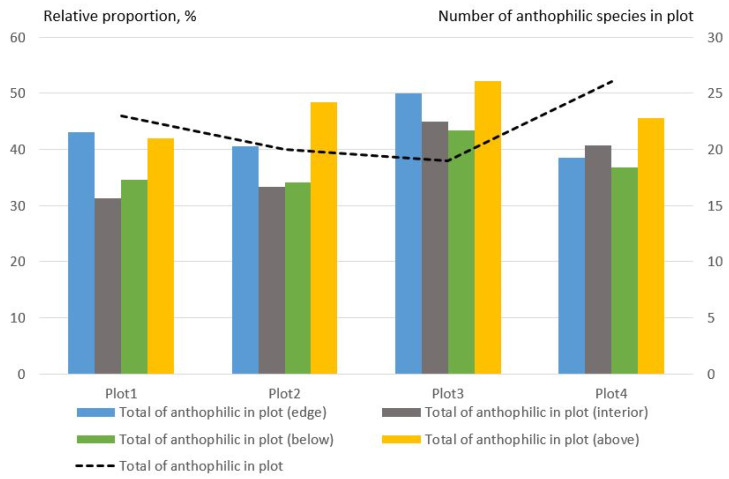
Relative proportion of anthophilic Coleoptera species collected using beer traps on the edges and in the forest interior (% of species per plot).

The number of anthophilic species in all traps was significantly lower than the number of saproxylic species. Their share varied in the different plots. However, on average, the number of anthophilic species on the edges was greater. All plots had a large proportion of anthophilic species at the edge in the upper traps (Figure 5).

**Figure 6:**
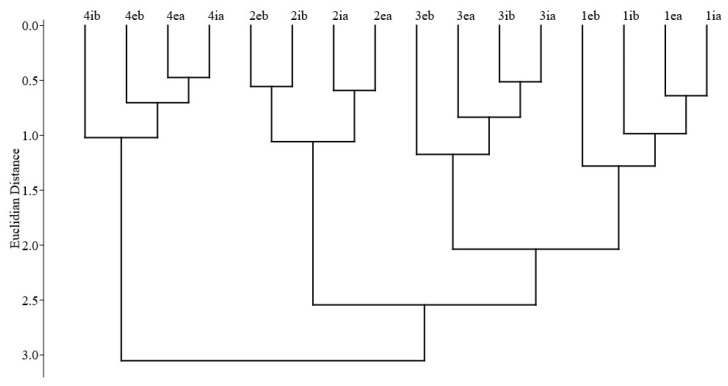
The similarity of the beetle species composition among all sampling variants (four sites, two height levels, and two positions regarding the proximity of the forest edge) based on the Jaccard index. We applied Ward’s method and the Euclidean distance (cophenetic correlation coefficient = 0.771). 1—plot1; 2—plot2; 3—plot3; 4—plot4; “i”—forest interior; “e”—edge of the forest; “a”—at the top height (7.5 m), “b”—at the lowest height (1.5 m).

According to the Jaccard index, the species composition differs most in the birch forest at plot4 (Figure 6). This plot forms a separate cluster. A second cluster is formed by three other plots. Within this cluster, the oak forests of plot3 and plot1 form a separate subcluster, while plot2 stands alone. Within each plot, it is possible to observe the grouping of samples according to the height (above—a, below—b) and position in the forest interior (i) and on the edge (e). In most cases, the samples were grouped as upper with upper and lower with lower, with the exception of plot3.

## 4. Discussion

This study provides data on the species diversity and abundance of Coleoptera sampled using bait beer traps set at two heights on edges and within deciduous forests of European Russia. Edges (transitional habitats and ecotones) have a major influence on the spatial distribution of many Coleoptera species [13,15,50,51]. Ecotones are transition zones that can contribute to biodiversity conservation, as they tend to be species-rich relative to neighboring habitats [52]. Determining the distance to which edge effects extend into deep forests is important to understand community biodiversity, the extent of habitat for rare species [50]. Some studies have shown that the invertebrate community composition gradually changes up to 400 m on either side of a boundary and that the distance to a marked change in the composition is dependent on one taxon or another [50,52]. Thus, the distances used in our studies from the edge into the forest interior provide a good indication of the differences among Coleoptera communities on the edges and in the forest interior.

The results show that Coleoptera species diversity is higher on the edges than in the forest interior. However, the total Coleoptera abundance is lower on the edges. In addition, the number of species in the lower traps is almost the same or higher than in the upper traps. Thus, there is a dependence in both the horizontal plane (edges–forest interior) and the vertical plane (below–above). Some differences in the results among the plots were obtained. In terms of the Jaccard’s index, the species composition differs the most in plot4. It is possible that a certain influence is exerted by the configuration of the forest area and the nearby open spaces of the fallow land. This is a birch forest, which is distinguished from other forests by the transparency of the crowns of the trees in the first tier. High solar insolation allows for a tier of shrubs and, especially, herbaceous plants to develop, forming a well-developed cover. The edge of this forest is transparent and allows many Coleoptera species to penetrate deep into the forest area. Opportunities for active Coleoptera to penetrate deep into woodlands have been described for many families [53,54,55,56]. It appears that the presence of herbaceous cover, which is to some extent similar to that in the nearby grassland ecosystem, provides Coleoptera with feeding and development opportunities. Apparently, this is why the Coleoptera abundance from plot4 is so high in the lower traps, both on the edges than in the forest interior. Among other things, it is in plot4 that the highest values of species diversity, total abundance, the Shannon index and the lowest values of the Simpson index were obtained.

In contrast to plot4, in another plot (plot3), the minimums of biodiversity, abundance, and Shannon index and a very high Simpson index were obtained. The agroecosystem is closely adjacent to this plot. There is practically no transition zone in the form of a meadow ecosystem between the edge and the agroecosystem. This is confirmed by the small number of anthophilic Coleoptera, whose number of species is much higher at the edge of the upper traps (these are species that mostly visit flowering shrubs). Apparently, this is why the total number of Coleoptera at the edge is lower than in the forest interior. There is also an interesting specificity: the number of Coleoptera at the edge and in the forest interior in the lower traps are almost the same, whereas in the upper traps in the forest interior, the number is actually twice as high than in similar traps located at the edge. The closeness of the crowns of the first tier trees in this plot is quite high, and little light penetrates into the lower tiers. Therefore, the number of Coleoptera in the forest interior increases in places that are illuminated by the Sun, i.e., in the crowns. This confirms the fact that the vertical stratification of insects in forests depends on the availability of the Sun/openness of the habitat [57]. The diversity of insect communities may also be lower or higher in the canopy than in the forest understory, depending on the canopy cover and/or the density of the understory [58,59].

Plot2 stands out as a separate subcluster. Apparently, in this case, the lack of horizontal connections among this glade and neighboring open spaces are influences. As a consequence, the Coleoptera fauna is represented by species that inhabit the glade itself and the surrounding forest. In this plot, a significant proportion of saproxylic Coleoptera in all traps was noted. As in plot4, greater species diversity and abundance were noted in the lower traps than in the upper traps. Plot1 had an increase in Coleoptera abundance in the upper traps, both at the edge and within the forest. However, an increase in the number of species was obtained in the lower traps. The number of anthophilic species was higher in the edge habitats, which can be explained by the considerable biodiversity of meadow grasses in the open ecosystem adjacent to the forest.

Only four species were found in all traps (*P. marmorata*, *C. strigata*, *G. grandis*, and *S. grisea*). Earlier, it was shown that *Protaetia marmorata* prefers tree crowns for its habitat and is more often found in traps in the first tier [22,60]. Apparently, in this case, this preference is influenced by the life cycle of the species, during which larval development occurs in the hollows of dead oaks and feeding of adults on sap flowing from tree trunks [61]. This species has also been shown to outnumber the plots at the edge than in the forest interior. According to our results, *Glischrochilus grandis* prefers the lower forest tiers inside forest patches (the abundance of this species is relatively lower on forest edges). A similar effect has been reported previously in deciduous forests [60]. This species is often found on decaying tree sap from birch and oak trees, on rotting berries, and on corkscrews. Its larvae develop on such decaying substrates [62,63]. Given that the larval development of *Glischrochilus grandis* occurs in places close to the soil surface and more often inside forests rather than on forest edges, the preference for lower forest tiers is understandable.

For the other two species (*C. strigata* and *S. grisea*), we note that different results have been shown for different plots. *Cryptarcha strigata* has previously been shown to occur slightly more frequently at 3.5 m in the forest interior. However, *Soronia grisea* was also found more frequently along with *Cryptarcha strigata* [60]. Both species are confined to oak, aspen, and mixed plantations with the presence of oak, where they often occur on the sap of *Q. robur*, *Salix* and *P. tremula* [63,64]. It is possible that the high activity of these species in different habitats, combined with some other unaccounted factors, influence the results.

Saproxilus beetles have been shown to account for 30% of all Coleoptera species in forest ecosystems [65]. Their species diversity is higher in warmer forest areas with an abundance of dead wood, stumps, deadwood, dead trees, and coarse woody debris [57,65,66,67,68]. It is clear that in this case most of the saproxylic beetles will be found inside the forest, as edge habitats and even more so open ecosystems are devoid of habitats for the life cycles of such species. This was exactly the case in our studies. Well-developed undergrowth and the growth of flowering shrubs, on which anthophiles find food, apparently explain the increase in the share of this group in the upper canopies.

Many factors can influence the response of insects in edges. For example, the orientation of the edge in relation to the Sun, the size and isolation of the habitats, the composition of the landscape, adjacent habitats, the permeability of the edge, soil characteristics, the species composition of trees and shrubs in the edge, seasonal phenomena, and habitat suitability can be responsible for unexplained changes in the research. Often, the impact of an edge forest is mediated by the history of the edge forest. Boundaries created by natural ecological processes are permeable to disturbance-sensitive species, but they limit the penetration of species from open habitats into the forest interior [3,69,70,71,72]. Our results showed that differences in the structure of the sampled woodlands and the influence of dominant tree species can explain the differences in the data obtained. However, when taken together, they provide quite definitive information for understanding the functioning of the communities and their biodiversity in edges and nearby forested areas. Overall, beetle abundance was influenced by the distance from the edge of the forest to the interior of the woodland and the height of the trap. In addition, we found significant differences in species composition among the study sites.

## 5. Conclusions

A total of 144 species from 35 families were identified in Coleoptera collections at edges and various heights in temperate forests of European Russia. In four plots, Cerambycidae, Nitidulidae, Curculionidae, and Elateridae had the highest species diversity. Common to all plots were 13 species, and only 4 of them were found in all traps (*Protaetia marmorata*, *Cryptarcha strigata*, *Glischrochilus grandis*, and *Soronia grisea*). Significant differences in species composition among the studied plots were obtained. These differences were due to the open ecosystems adjacent to the plots, the crowning of the upper layer, the presence of the second and herbaceous layer, and the possibility of an edge for species to penetrate deep into the forest. A common pattern was the highest Coleoptera species diversity at the edges in the lower traps. However, the total abundance of all species was lower at the edges. The Shannon index was almost always higher or equal in the traps located in the forest interior. Based on the mean values from all plots, the number of saproxylic Coleoptera species was found to be greater inside the forest plots, and the highest number of saproxylic species was found in the upper traps. For all plots, a greater number of anthophilic species were obtained at the edge of the forest in the upper traps.

## Figures and Tables

**Table 1:** The main indicators of the number and species diversity of Coleoptera collected using beer traps on the edges and in the forest interior.

Species	Plot1				Plot2				Plot3				Plot4			
	Edge		Interior		Edge		Interior		Edge		Interior		Edge		Interior	
	Below	Above	Below	Above	Below	Above	Below	Above	Below	Above	Below	Above	Below	Above	Below	Above
Total number of individuals	311	717	422	458	712	564	1216	853	494	438	552	1016	2128	683	1807	671
Number of species (excluding unidentified ones)	41	25	24	18	28	24	33	22	23	20	17	13	42	36	43	36
Number of saproxylic species (% of the total number of species per plot)	46.3	84.0	70.8	72.2	89.3	83.3	75.8	81.8	65.2	80.0	88.2	84.6	66.7	61.1	65.1	69.4
Number of anthophilic species (% of the total number of species per plot)	41.5	48.0	37.5	27.8	35.7	50.0	33.3	40.9	39.1	60.0	47.1	38.5	40.5	52.8	37.2	47.2
Total number of families	14	10	13	9	12	8	14	8	12	8	8	5	16	12	18	18
Shannon index	2.52	1.92	2.23	1.79	1.41	1.43	1.92	1.6	1.06	1.23	1.21	1.07	2.12	2.41	1.91	2.09
Simpson index	0.14	0.20	0.16	0.23	0.47	0.39	0.23	0.29	0.58	0.52	0.45	0.52	0.17	0.16	0.22	0.19
Total number of individuals per plot	1908				3345				2500				5289			
Total number of individuals per plot (edge)	1028				1276				932				2811			
Total number of individuals per plot (interior)	880				2069				1568				2478			
Total number of individuals per plot (below)	733				1928				1046				3935			
Total number of individuals per plot (above)	1175				1417				1454				1354			
Shannon index per plot	2.36				1.91				1.20				2.29			
Simpson index per plot	0.14				0.29				0.50				0.15			
Total number of families per plot	20				19				16				28			

## Data Availability

The data presented in this study are available on request from the corresponding author.

## Appendix A

**Table A1:** Species diversity and absolute number of Coleoptera individuals collected using beer traps at different sites in the forests of the center of European Russia.

Species	Plot1				Plot2				Plot3				Plot4			
	Edge		Interior		Edge		Interior		Edge		Interior		Edge		Interior	
	Below	Above	Below	Above	Below	Above	Below	Above	Below	Above	Below	Above	Below	Above	Below	Above
**Carabidae**																
*Agonum lugens* (Duftschmid, 1812)	1															
*Amara tibialis* (Paykull, 1798)	1															
*Calathus fuscipes* (Goeze, 1777)	1															
*Calosoma inquisitor* (Linnaeus, 1758)			9													
*Carabus cancellatus* Illiger, 1798															1	
*Harpalus rufipes* (De Geer, 1774)									1							
*Limodromus assimilis* (Paykull, 1790)			5													
**Scirtidae**																
*Contacyphon pubescens* (Fabricius, 1792)																1
*Contacyphon* sp.							1	1								
**Buprestidae**																
*Agrilus sulcicollis* (Lacordaire, 1835)									1							
**Throscidae**																
*Trixagus* sp.									1		2		1			
**Cantharidae**																
*Cantharis livida* (Linnaeus, 1758)										1			9	38		
*Cantharis nigricans* (O.F. Müller, 1776)							1	1					1	2		3
*Cantharis figurata* (Mannerheim, 1843)	2	1														
*Cantharis pellucida* (Fabricius, 1792)													4	11		1
*Rhagonycha lignosa* (O.F. Müller, 1764)	1															
*Rhagonycha nigriventris* (Motschulsky, 1860)														1		
**Elateridae**																
*Agrypnus murinus* (Linnaeus, 1758)													3	1	1	
*Ampedus nigroflavus* (Goeze, 1777)														2		
*Ampedus pomorum* (Herbst, 1784)														2		
*Ampedus sanguinolentus* (Schrank, 1776)	2													3		
*Athous haemorrhoidalis* (Fabricius, 1801)		1	2	1												
*Athous subfuscus* (O.F. Müller, 1764)							1									
*Dalopius marginatus* (Linnaeus, 1758)													2	1	1	1
*Denticollis borealis* (Paykull, 1800)													1		1	
*Denticollis linearis* (Linnaeus, 1758)							1									
*Prosternon tesselatum* (Linnaeus, 1758)	6					3							5	5	3	
*Selatosomus aeneus* (Linnaeus, 1758)														2		
**Lampyridae**																
*Lampyris noctiluca* (Linnaeus, 1758)							2									
**Histeridae**																
*Eurosomides minor* (P. Rossi, 1792)					1											
*Gnathoncus buyssoni* (Auzat, 1917)								1								
*Hister unicolor* (Linnaeus, 1758)			1										1			
*Margarinotus striola* (C.R. Sahlberg, 1819)					8		11						10	2	13	2
*Platysoma elongatum* (Thunberg, 1787)															1	
*Platysoma lineare* Erichson, 1834							1									
*Saprinus rugifer* (Paykull, 1809)	1															
**Staphylinidae**																
Staphylinidae sp.	18	12	32	12	39	8	90	27	30	1	13	3	211	21	98	22
*Dendroxena quadrimaculata* (Scopoli, 1771)						1		2							1	
*Necrodes littoralis* (Linnaeus, 1758)					1		2			2		2	1			
*Nicrophorus vespilloides* Herbst, 1783																
*Oiceoptoma thoracicum* (Linnaeus, 1758)	2	1	14	1			9						27		17	1
*Quedius dilatatus* (Fabricius, 1787)				1	1	2	27	158								
**Scarabaeidae**																
*Cetonia aurata* (Linnaeus, 1758)	2	2				5	1			5	2	2		5	1	1
*Gnorimus variabilis* (Linnaeus, 1758)		1				1										
*Protaetia fieberi* (Kraatz, 1880)	3	13	3			13			1	11	1	10	2	10		11
*Protaetia marmorata* (Fabricus, 1792)	4	127	3	87	18	124	3	41	1	10	1	27	44	187	40	150
*Protaetia speciosissima* (Scopoli, 1786)														1		1
*Protaetia cuprea volhyniensis* (Gory and Percheron, 1833)					1	5				2		1	8	7		3
*Serica brunnea* (Linnaeus, 1758)	1						1									
**Dermestidae**																
*Attagenus schaefferi* (Herbst, 1792)													1	4	1	1
*Ctesias serra* (Fabricius, 1792)								2					2	2		2
*Megatoma undata* (Linnaeus, 1758)													1	7	3	2
*Trogoderma glabrum* (Herbst, 1783)	1									1			15	4		1
**Ptinidae**																
*Dorcatoma robusta* (A. Strand, 1938)							1									
*Dorcatoma flavicornis* (Fabricius, 1792)										1						
*Ptinus villiger* (Reitter, 1884)				1					1							
**Byturidae**																
*Byturus ochraceus* (L.G. Scriba, 1790)											1					
**Trogossitidae**																
*Peltis grossa* (Linnaeus, 1758)																1
**Melyridae**																
*Dasytes fusculus* (Illiger, 1801)															2	
*Dasytes niger* (Linnaeus, 1761)	14		9		2											
*Dolichosoma lineare* (P. Rossi, 1794)	5															
*Malachius bipustulatus* (Linnaeus, 1758)	1														1	
**Lymexylidae**																
*Elateroides dermestoides* (Linnaeus, 1761)															1	
**Mordellidae**																
*Mordellistena* sp.	1															
*Tomoxia bucephala* (A. Costa, 1854)					1											
*Variimorda* sp.	1															
**Scraptiidae**																
*Anaspis frontalis* (Linnaeus, 1758)															1	1
**Oedemeridae**																
*Oedemera femorata* (Scopoli, 1763)	2								1							
*Oedemera virescens* (Linnaeus, 1767)	1															
**Melandryidae**																
*Orchesia micans* (Panzer, 1793)					1											
**Zopheridae**																
*Synchita humeralis* (Fabricius, 1792)		1														1
**Mycetophagidae**																
*Litargus connexus* (Geoffroy, 1785)			1				1				2	2	1		2	1
*Mycetophagus piceus* (Fabricius, 1777)																1
*Mycetophagus quadripustulatus* (Linnaeus, 1761)											1					
**Tenebrionidae**																
*Mycetochara flavipes* (Fabricius, 1792)															1	
*Upis ceramboides* (Linnaeus, 1758)					1											
**Cerylonidae**																
*Cerylon ferrugineum* (Stephens, 1830)									1				1			
**Coccinellidae**																
*Calvia decemguttata* (Linnaeus, 1767)							1	1						1		
*Chilocorus renipustulatus* (L.G. Scriba, 1791)			1													
*Coccinella magnifica* L. (Redtenbacher, 1843)					2											
*Coccinella septempunctata* (Linnaeus, 1758)									1							
*Halyzia sedecimguttata* (Linnaeus, 1758)															1	
*Hyperaspis concolor* (Suffrian, 1843)	1															
*Nephus bipunctatus* (Kugelann, 1794)													2			
*Psyllobora vigintiduopunctata* (Linnaeus, 1758)	1		1												1	
**Erotylidae**																
*Dacne bipustulata* (Thunberg, 1781)													1			
*Triplax russica* (Linnaeus, 1758)													1		2	
**Monotomidae**																
*Rhizophagus fenestralis* (Linnaeus, 1758)						1	2						6		11	3
*Rhizophagus picipes* (G.-A. Olivier, 1790)			1													
**Nitidulidae**																
*Carpophilus hemipterus* (Linnaeus, 1758)		1				1										
*Carpophilus* sp.													1			
*Cryptarcha strigata* (Fabricius, 1787)	9	201	32	161	442	321	402	375	322	290	319	702	407	151	141	200
*Cryptarcha undata* (G.-A. Olivier, 1790)	3	45		9	29	28	7	4	6	36	19	80	23	24	16	26
*Cychramus luteus* (Fabricius, 1787)	1		1		16	1	183	20			1					
*Cychramus variegatus* (Herbst, 1792)					1		4									
*Epuraea guttata* (G.-A. Olivier, 1811)	1	2		2	9	1	3	3	13	3	6	5	6	4	1	1
*Epuraea* sp.	12	21	31	11	15	4	39	9	36	25	25	6	81	8	90	8
*Glischrochilus grandis* (Tournier, 1872)	35	10	50	8	8	1	28	12	28	6	27	8	489	36	466	32
*Glischrochilus hortensis* (Geoffroy, 1785)	60	6	64	17	27	5	267	162			5	19	292	12	472	33
*Glischrochilus quadriguttatus* (Fabricius, 1777)		8	7	16	2		28	2								
*Glischrochilus quadripunctatus* (Linnaeus, 1758)					1		32	1					4		8	
*Glischrochilus quadrisignatus* (Say, 1835)	3							1	3	1			3		2	
*Ipidia binotata* (Reitter, 1875)	1														1	
*Meligethes* sp.									1						1	
*Omosita japonica* (Reitter, 1874)	1															
*Soronia grisea* (Linnaeus, 1758)	43	166	33	83	66	22	29	19	34	35	117	148	113	15	37	19
*Soronia punctatissima* (Illiger, 1794)													6			
**Cryptophagidae**																
*Cryptophagus* sp.																1
**Cucujidae**																
*Cucujus haematodes* (Erichson, 1845)			1													
*Pediacus depressus* (Herbst, 1797)		1			1	1	2						1	3		1
**Cerambycidae**																
*Anoplodera sexguttata* (Fabricius, 1775)											1					
*Aromia moschata* (Linnaeus, 1758)	1	7								2						
*Dinoptera collaris* (Linnaeus, 1758)							1									
*Leptura quadrifasciata* Linnaeus, 1758		1			13	6	8	2	4	1	5		2		3	
*Leptura thoracica* Creutzer, 1799					2	5	2	4	1	1			2	22	5	13
*Lepturalia nigripes* (De Geer, 1775)									1							
*Mesosa myops* (Dalman, 1817)													1			
*Necydalis major* Linnaeus, 1758		1		1												
*Obrium cantharinum* (Linnaeus, 1767)								1		1						
*Phymatodes testaceus* (Linnaeus, 1758)															1	
*Plagionotus detritus* (Linnaeus, 1758)										2						
*Purpuricenus kaehleri* (Linnaeus, 1758)	1	4														
*Rhagium mordax* (De Geer, 1775)		3	7		1	1	23	3					67	19	39	8
*Rhagium sycophanta* (Schrank, 1781)			1		1	1		1								
*Stenocorus meridianus* (Linnaeus, 1758)				1		2										
*Stictoleptura maculicornis* (De Geer, 1775)																1
*Strangalia attenuata* (Linnaeus, 1758)									1							
*Xylotrechus rusticus* (Linnaeus, 1758)																1
**Chrysomelidae**																
*Galerucella pusilla* (Duftschmid, 1825)				1												
*Hypocassida subferruginea* (Schrank, 1776)						1										
*Lochmaea caprea* (Linnaeus, 1758)	1															
*Phyllotreta vittula* (L. Redtenbacher, 1849)														1		
*Phratora atrovirens* (Cornelius, 1857)															1	
*Plagiodera versicolora* (Laicharting, 1781)															1	
**Anthribidae**																
*Gonotropis dorsalis* (Gyllenhal, 1813)				1												
*Platystomos albinus* (Linnaeus, 1758)															1	
*Tropideres albirostris* (Schaller, 1783)	3	1														
**Attelabidae**																
*Deporaus betulae* (Linnaeus, 1758)																1
**Curculionidae**																
*Anisandrus dispar* (Fabricius, 1792)	58	79	106	43	1						3		261	63	310	112
*Anthonomus rubi* (Herbst, 1795)		1														
*Archarius pyrrhoceras* (Marsham, 1802)									1							
*Brachysomus echinatus* (Bonsdorff, 1785)												1				
*Cleopomiarus distinctus* (Boheman, 1845)	1															
*Mononychus punctumalbum* (Herbst, 1784)	1															
*Phyllobius argentatus* (Linnaeus, 1758)							1									
*Phyllobius pomaceus* Gyllenhal, 1834	1		2	1												
*Phyllobius pyri* (Linnaeus, 1758)	1												2			
*Polydrusus cervinus* (Linnaeus, 1758)									1				2	1		2
*Polydrusus mollis* (Strøm, 1768)									1	1	1				1	
*Polydrusus tereticollis* (De Geer, 1775)									1				4	3	4	1
*Strophosoma capitatum* (De Geer, 1775)					1		1							2	1	
*Xyleborinus saxesenii* (Ratzeburg, 1837)	1		5						1							

The family names are marked in bold.
